# Direct Medical Cost of Stroke and the Cost-Effectiveness of Direct Oral Anticoagulants in Atrial Fibrillation-Related Stroke: A Cross-Sectional Study

**DOI:** 10.3390/ijerph19031078

**Published:** 2022-01-19

**Authors:** Siti Norain Azahar, Saperi Sulong, Wan Asyraf Wan Zaidi, Norliza Muhammad, Yusof Kamisah, Norliana Masbah

**Affiliations:** 1 Department of Pharmacology, Faculty of Medicine, Universiti Kebangsaan Malaysia, Jalan Yaacob Latif, Kuala Lumpur 56000, Malaysia; drainazahar@gmail.com (S.N.A.); norliza_ssp@ppukm.ukm.edu.my (N.M.); kamisah_y@ppukm.ukm.edu.my (Y.K.); 2 Department of Community Health, Faculty of Medicine, Universiti Kebangsaan Malaysia, Kuala Lumpur 56000, Malaysia; saperi@ppukm.ukm.edu.my; 3 Neurology Unit, Department of Internal Medicine, Faculty of Medicine, Universiti Kebangsaan Malaysia, Kuala Lumpur 56000, Malaysia; wan.asyraf.wan.zaidi@ppukm.ukm.edu.my

**Keywords:** stroke, atrial fibrillation, direct medical cost, direct oral anticoagulants, cost-effectiveness analysis

## Abstract

Background: Stroke has significant direct medical costs, and direct oral anticoagulants (DOACs) are better alternatives to warfarin for stroke prevention in atrial fibrillation (AF). This study aimed to determine the direct medical costs of stroke, with emphasis on AF stroke and the cost-effectiveness of DOACs among stroke patients in a tertiary hospital in Malaysia. Methods: This study utilised in-patient data from the case mix unit of Universiti Kebangsaan Malaysia Medical Centre (UKMMC) between 2011 and 2018. Direct medical costs of stroke were determined using a top-down costing approach and factors associated with costs were identified. Incremental cost effectiveness ratio (ICER) was calculated to compare the cost-effectiveness between DOACs and warfarin. Results: The direct medical cost of stroke was MYR 11,669,414.83 (n = 3689). AF-related stroke cases had higher median cost of MYR 2839.73 (IQR 2269.79–3101.52). Regression analysis showed that stroke type (AF versus non-AF stroke) (*p* = 0.013), stroke severity (*p* = 0.010) and discharge status (*p* < 0.001) significantly influenced stroke costs. DOACs were cost-effective compared to warfarin with an ICER of MYR 19.25. Conclusions: The direct medical cost of stroke is substantial, with AF-stroke having a higher median cost per stroke care. DOACs were cost effective in the treatment of AF-related stroke in UKMMC.

## 1. Introduction

Stroke is defined as the development of rapid clinical signs due to disturbed cerebral functions which originated from a vascular source [1]. According to the Global Burden of Disease Study, stroke accounted for 5.5 million deaths and a staggering 116.4 million disability adjusted life years (DALYs) in 2016 [2]. A large proportion of stroke cases are cardioembolic in origin, with atrial fibrillation (AF) accounting for 25% of acute ischaemic strokes in elderly patients [3]. AF is a prevalent form of arrythmia which increases stroke risk between three- and four-fold [4], and is increasingly common in individuals older than 80 years old. AF is known to be paroxysmal and often asymptomatic. Therefore, it is not surprising that its first clinical manifestation may present as a stroke episode. 

A similar burden of disease is also seen in the vastly populated Asian countries. By 2050, 72 million AF patients are projected in Asia, with almost 2.9 million of them suffering from AF-related stroke [5]. In Malaysia, the prevalence of stroke has increased from 0.3% in 2006 [6], to 0.7% in 2011 [7]. Meanwhile, in terms of AF figures, the Malaysian Responding to Increasing Cardiovascular (REDISCOVER) disease prevalence study has reported a prevalence of 0.54% in 2007, which is considered relatively low compared to the worldwide AF prevalence of 1% [8]. However, with the nation shifting towards aging population, it is anticipated that the prevalence of AF will continue to rise. Ischaemic stroke is the commonest aetiology seen in AF-related stroke patients, which is often associated with higher costs and a more severe prognosis [9]. AF-related strokes tend to be more severe due to a higher mortality risk and increased disability rate in patients, resulting in longer hospital stays with higher direct costs of stroke care [10]. Based on these factors, it is not surprising that the established economic burden of stroke may be further exacerbated by concomitant presence of AF. The economic burden of stroke alone is enormous, consuming approximately 3–4% of total healthcare expenditure in Western countries [11]. With the presence of AF, stroke costs can increase significantly. A number of Western studies have drawn attention to the significantly increased cost burden of AF-related ischaemic strokes, whereby AF stroke costs recorded were substantially higher than non-AF stroke cases [12,13,14]. To date, very few Malaysian studies have explored the direct medical costs of stroke [15,16,17]; however, no emphasis has been placed on investigating the direct medical costs incurred by AF ischaemic stroke to the healthcare provider thus far. 

Due to the substantial cost burden of AF-related strokes, stroke prevention in AF patients is imperative. Long term oral anticoagulant therapy has been the mainstay of treatment for AF-related stroke prevention for decades. Aside from its high cost burden, AF is considered a poor predictor of clinical outcome in patients with ischaemic stroke, thus stroke prevention is critical in this patient cohort. Clinical trials have shown that vitamin K antagonists (VKAs) such as warfarin can reduce stroke risk by up to 64% compared to the use of control or placebo [18]. However, although warfarin has proven to be an effective anticoagulant for decades, it has significant drawbacks, such as a narrow therapeutic index, numerous food and drug interactions, and requires cumbersome laboratory monitoring and dose adjustment to maintain the international normalised ratio (INR) of blood level within the therapeutic range [19]. Therefore, routine INR monitoring associated with warfarin therapy incurs greater costs to the healthcare system [20].

A decade ago, in 2011, alternative treatments for AF-stroke prevention came to light. They are known as direct oral anticoagulants (DOACs), which consist of dabigatran etexilate, rivaroxaban, apixaban and edoxaban. Over the years, a number of large-scale clinical trials have shown the superiority of DOACs over warfarin in terms of stroke incidence and systemic embolism, with a lower risk of major bleeding especially for apixaban and low-dose dabigatran [21,22,23]. Unlike warfarin, DOACs target a specific coagulation factor (factor Xa or thrombin) directly, therefore providing a much faster anticoagulant effect. Because DOACs can be administered in fixed doses without the need for routine INR monitoring or dietary restrictions [24], it confers a greater advantage in terms of patient’s compliance and adherence. Similar to the fact that the economic burden of strokes (particularly AF-related strokes) is under-explored in developing Asian countries, the cost-effectiveness of DOACs compared to warfarin has yet to gain its spotlight in this region. In the last decade, there have been few studies from Asia looking at the cost-effectiveness of DOACs compared to warfarin for AF stroke prevention, and most of them investigated the cost-effectiveness of DOACs using a hypothetical economic modelling approach [25,26,27,28,29]. Given the substantial economic burden of stroke and the limited availability of local data on the cost-effectiveness of DOACs for AF-related stroke prevention in Malaysia, there is an urgent need to determine the direct medical costs of stroke, particularly in the presence of AF, using secondary data from a tertiary hospital in Kuala Lumpur, Malaysia. Subsequently, the factors influencing the direct medical costs of stroke were identified in this patient cohort. We also investigated the cost-effectiveness of DOACs compared with warfarin in this study population. 

## 2. Materials and Methods

### 2.1. Study Setting and Design

This is a retrospective, cross-sectional study which utilised secondary data maintained by the case mix Unit of a tertiary level hospital in Kuala Lumpur, the Universiti Kebangsaan Malaysia Medical Centre (UKMMC). The data consisted of inpatient records of stroke patients who were hospitalised in UKMMC from year 2010 to 2018. The study protocol was approved by the UKM research ethics committee, UKMMC (FF-2018-451; ethics approval code JEP-2018-641). Case mix data comprises of discharged data of patients who were hospitalised, categorised according to their diagnosis based on the International Classification of Diseases 10th revision (ICD-10) coding system. The case mix system uses the tool or grouper developed by the United Nations University International Institute for Global Health (UNU-IIGH) and the National University of Malaysia (UKM). The system has been implemented in UKMMC since 2002 and is based on top-down costing method. Variables included in the case mix data are length of stay, the tariff (hospital costs incurred) per patient, stroke subtypes (ischaemic or haemorrhagic), severity of illness, comorbidities, procedures, discharged status and other key demographics. In this study, the case mix data were used instead of the usual hospital discharge data because the patients’ diagnoses were recorded based on the International Classification of Diseases 10th revision (ICD-10) coding system, which allows categorical classification of cases based on systematic codes. 

### 2.2. Recruitment of Study Sample

This study involved universal sampling method, in which all patients with a primary diagnosis of stroke who were admitted at UKMMC from 2011 until 2018 were enrolled as participants. Patients who were coded under ICD-10 (10th revision) coding system with codes 161, I63, and I64 (cerebrovascular diseases) were included in the study. Other inclusion criteria for the study include male and female patients who were at least 18 years of age that were hospitalised due to stroke as the main diagnosis. In addition to that, patients who had concomitant atrial fibrillation (either newly diagnosed at index event or prior to index stroke), with varying degree of stroke severity were recruited. Patients were identified as having AF if there was any documentation of AF in their case notes within the recent years, if AF was newly diagnosed during the ECG testing at the time of their ward admission, or if AF was picked up on prolonged cardiac monitoring in the ward after the stroke event. 

Exclusion criteria include patients who were under 18 years of age and stroke patients who were admitted with longer length of hospitalisation due to other comorbidities which are not related to stroke as the primary diagnosis (for example chronic renal failure, or respiratory diseases such as chronic obstructive pulmonary disease). Subsequently, the subjects were categorised according to the aetiology of stroke (ischaemic or haemorrhagic) and therefore were stratified based on the presence of AF to determine the specific AF stroke-related costs. 

### 2.3. Data Collection

Apart from the principal data obtained from the case mix unit, information on the types and the cost of anticoagulants prescribed to the stroke patients during the study period were obtained from Medipro software database system in the Pharmacy Department, UKMMC. Additional information on the subjects recruited were obtained from patients’ medical case notes which were retrieved from the Research and Statistics Unit, Department of Health Information UKMMC. This additional information consisted of the baseline stroke severity during initial admission (for AF ischaemic stroke subjects). The stroke severity was based on the modified Rankin score (mRS) recorded in the hospital case notes during inpatient admission, as documented by the physician in-charge during their hospital stay. The risk of stroke among the subgroup of AF patients in particular was determined using the CHADS2 scoring system. The CHADS2 (Congestive heart failure, Hypertension, Age ≥ 75 years, Diabetes, previous Stroke (double weight)) score is a simple stroke risk stratification tool to predict stroke risk and identify high-risk patients who will benefit the most from anticoagulant medications, thereby improving patient outcomes.

### 2.4. Calculation of Direct Medical Cost of Stroke 

The calculation for direct medical cost of stroke was performed for years 2011 to 2018. Data on resource use and cost evaluation that was collected retrospectively was based on case mix costing, which involved the actual direct costs analysis mainly based on inpatient LOS. In the costing process, direct costs usually represent the costs associated with medical resource utilization and all activities which reflect the workload starting from patient’s admissions, duration of stay in the ward (length of stay), laboratory and radiological tests performed and prescription of pharmacy within the health care delivery system. In this study, the direct medical costs of stroke from 2011–2018 was determined using a top-down costing approach, taken from the health care provider’s perspective. The top-down costing approach involved analysis of the hospital’s operational costs associated with multiple levels of cost centres in UKMMC. Case mix system adopts a top-down costing approach, beginning with the total hospital expenditures that are then divided by different homogenous patient groups with clinically similar characteristics and outcomes that are expected to use similar amounts of hospital resources. The total hospital expenditures for a specific year are divided into several cost centres to obtain the final unit cost, which is the cost of providing services for each patient per day of stay. Cost centres in UKMMC comprise three cost centres, namely the overhead cost centres, intermediate and final cost centres [16]. Overhead costs centres involve administration, utilities, consumables as well as services such as laundry, food, and dietetic services. The intermediate cost centres comprise of the charges for laboratory, radiology, pharmacy, and operating theatres. The final cost centres consist of the hospital wards and outpatient clinics [16]. To obtain the cost of care per patient per admission, this unit cost will be multiplied with the individual patient’s length of stay. However, the case mix data has automatically calculated the unit cost and multiplied this unit cost with individual patient’s LOS, which is derived in the form of the case mix tariff. The case mix tariff is essentially the total cost of care per patient per admission. Therefore, the direct medical cost of stroke for this study was derived directly from the tariff provided in the case mix database.

### 2.5. Cost-Effectiveness Analysis (CEA) of DOACS

Cost effectiveness of DOACs in the treatment of AF-related stroke was determined based on the calculation of cost-effectiveness ratio (CER) and incremental cost-effectiveness ratio (ICER). CEA of DOACs was conducted not only based on the drug acquisition costs of the two treatment modalities (DOACs versus warfarin), but also in terms of treatment outcomes obtained from each of the modalities used. In the context of this study, the outcome evaluated was stroke severity among AF ischaemic stroke (IS) patients in UKMMC, based on their mRS scores. CER was obtained by dividing the total cost of an intervention with their units of effectiveness as was shown in the formula below [30]:

$$\mathrm{CER}=\frac{\mathrm{Total}\;\mathrm{cost}}{\mathrm{Unit}\;\mathrm{of}\;\mathrm{effectiveness}}$$

Hence, based on the formula, the CER calculation for DOACs in this study is as follows:

$$\mathrm{CER}=\frac{\mathrm{cost}\;\mathrm{of}\;\mathrm{DOACs}\left(\mathrm{new}\;\mathrm{option}\right)\;\mathrm{or}\;\mathrm{cost}\;\mathrm{of}\;\mathrm{warfarin}\;\left(\mathrm{standard}\;\mathrm{option}\right)}{\mathrm{outcome}\;\mathrm{of}\;\mathrm{DOACs}\;\left(\mathrm{new}\;\mathrm{option}\right)\mathrm{or}\;\mathrm{outcome}\;\mathrm{of}\;\mathrm{warfarin}\;\left(\mathrm{standard}\;\mathrm{option}\right)}$$

Meanwhile, the calculated ICER facilitates comparison of cost-effectiveness between the two interventions from the economic perspective based on the per capita gross domestic product (GDP) of the country [31]. According to Shi and Nambudiri (2017) [32], the basic formula for the ICER of an intervention X relative to a comparison intervention Y (the current standard treatment) is as follows:

$$\mathrm{ICER}=\frac{\mathrm{Cost}\;\mathrm{of}\;\mathrm{intervention}\;X\;\left(\mathrm{new}\;\mathrm{option}\right)-\mathrm{cost}\;\mathrm{of}\;\mathrm{comparison}\;\mathrm{intervention}\;Y\;\left(\mathrm{standard}\;\mathrm{option}\right)}{\mathrm{Outcome}\;\mathrm{of}\;\mathrm{intervention}\;X\;\left(\mathrm{new}\;\mathrm{option}\right)-\mathrm{outcome}\;\mathrm{of}\;\mathrm{comparison}\;\mathrm{intervention}\;Y\;\left(\mathrm{standard}\;\mathrm{option}\right)}$$

The result is the incremental cost per unit of benefit gained. In the context of this study, the calculation of ICER is based on the cost of DOACs, in comparison to the cost of warfarin and the outcomes (benefits) gained from those two treatment options. In this study, we defined the outcome as the severity of stroke experienced by AF ischaemic stroke patients, which was translated as the percentage of AF ischaemic stroke patients with mild degree of stroke (mRS score of 0–2). Therefore, for this study,

$$\mathrm{ICER}=\frac{\mathrm{Cost}\;\mathrm{of}\;\mathrm{DOACs}-\mathrm{cost}\;\mathrm{of}\;\mathrm{warfarin}}{\mathrm{Outcome}\;\left(\mathrm{effectiveness}\right)\mathrm{of}\;\mathrm{DOACs}-\mathrm{outcome}\;\left(\mathrm{effectiveness}\right)\mathrm{of}\;\mathrm{warfarin}}\;=\frac{\mathrm{Cost}\;\mathrm{of}\;\mathrm{DOACs}-\mathrm{cost}\;\mathrm{of}\;\mathrm{warfarin}}{\%\;\mathrm{of}\;\mathrm{AF}\;\mathrm{IS}\;\mathrm{on}\;\mathrm{DOACs}\;\mathrm{with}\;\mathrm{mild}\;\mathrm{stroke}\;\left(\mathrm{mRS}\;0-2\right)-\%\;\mathrm{of}\;\mathrm{AF}\;\mathrm{IS}\;\mathrm{on}\;\mathrm{warfarin}\;\mathrm{with}\;\mathrm{mild}\;\mathrm{stroke}\;\left(\mathrm{mRS}\;0-2\right)}$$

### 2.6. Statistical Analysis

Statistical analysis was performed using SPSS version 23.0. Descriptive analysis was used for categorical data of patients. Mann–Whitney U test was used for comparison of stroke care cost and length of stay (LOS) between stroke types (AF and non-AF-related stroke), and types of oral anticoagulant used among the patients (NOACs vs. warfarin). Other factors which have influenced the direct medical cost of stroke such as LOS, stroke subtypes, stroke severity level (based on mRS score), and patient’s discharge disposition status were analysed using Kruskal–Wallis test. Statistical significance was set at *p* values < 0.05. Variables that have statistically significant association with direct medical costs based on the univariate analysis were subsequently entered into multiple linear regression analysis to estimate their magnitude of influence on direct medical cost of stroke for the cohort of this study.

## 3. Results

### 3.1. Patient Characteristics

#### 3.1.1. Total Stroke Patients (AF-Related Stroke and Non-AF Related Stroke)

A total of 3689 stroke patients were recorded in the case mix database for year 2011 until 2018. Key demographics of the study subjects are shown in Table 1. AF related stroke accounted for 9.3% of the total cases. The majority of the aetiology for stroke cases was ischaemic in origin (62.5%). Nearly half of the patients had a mild degree of stroke, with mRS score of 0–2 (45.9%), followed by a moderate degree of stroke (mRS score of 3–4) (36%), and severe stroke (mRS score of 5) (9.6%). The average length of stay was 5 ± 3.4 days, with a large proportion of patients staying between 4 and 7 days (42.2%) and 26.5% of them stayed more than 7 days.

#### 3.1.2. Subgroup of AF-Related Stroke Patients 

Table 2 summarises the characteristics of AF-related stroke patients. The mean age for AF-related stroke group was older compared to the mean age (SD) for overall cohort (71 ± 9.8 years). The AF-related stroke group had slightly longer average LOS compared to the mean for overall cohort, which was 6 ± 4.2 days. More than half of the AF-related stroke patients had a CHADS2 score of 2 or more (54.7%). Table 3 highlights the oral anticoagulant profile received by AF-related stroke patients. As shown in Table 3, most AF-related stroke patients were not on any baseline anticoagulants (80.2%) prior to hospitalisation for index stroke. Only a small proportion were on oral anticoagulants, with 12.2% were on warfarin, and 7.3% on DOACs. For patients who were on DOACs, the majority of them were on dabigatran 110 mg (32%), followed by rivaroxaban 15 mg (20.0%), dabigatran 150 mg (16%), rivaroxaban 20 mg and apixaban 5.0 mg (12.0%, respectively) and apixaban 2.5 mg (8%). However, anticoagulant prescription improved by 27.6% following hospitalisation, in which the percentage of patients who were prescribed with DOACs were increased to 25.6%, with dabigatran 150 mg use dominated other DOACs (25%), followed by warfarin (19.5%) after the stroke event.

### 3.2. Direct Medical Cost of Stroke Evaluation

Over the duration of 7 years (2011 to 2018), the total direct medical costs for all stroke cases (n = 3689) were MYR 11,669,414.83. The median direct medical cost of stroke (median, interquartile range, IQR) per patient per admission or per episode of stroke care is MYR 2269.79 (2269.79–2924.96). Meanwhile, the median cost of stroke treatment per patient per inpatient day (in the ward) for the overall cohort of patient is MYR 453.96. The total direct medical costs and the median cost per patient per admission for the AF-related stroke group and non-AF stroke subjects are described in Table 4.

Table 5 shows the comparison of length of stay, median cost and median mRS score for AF ischaemic stroke patients who were on oral anticoagulants (either DOACs or warfarin) during hospitalisation (n= 155). The median direct medical cost of AF related stroke patients who were on DOACs was higher compared to those on warfarin (MYR 2839.73 vs. MYR 2269.79). However, the median LOS for AF related stroke patients who had received DOACs was shorter (5 days) compared to those patients in the warfarin group (7 days).

Table 6 summarises the results of the standard multiple regression analysis in the overall study patients. For the total stroke cases, both stroke types and patients’ LOS had significantly influenced the direct medical cost of stroke. It was found that cost of care for AF related stroke was MYR 274.57 higher than non-AF related stroke and this effect was significant (*p* = 0.013). Analysis also showed a significant difference in terms of patients’ LOS, with those who had stayed and were treated in the hospital more than 7 days had cost of stroke care of MYR 2158.01 much higher than those with LOS of 3 days or less (*p* = 0.001).

Table 7 shows the standard multiple regression analysis for cost of stroke care in AF-related stroke patients. The regression model is significant for all 3 predictors, as follows: stroke subtypes, stroke severity level, and patients’ discharge disposition status. Patients with haemorrhagic stroke had cost of care of MYR 1514.27, much higher than ischemic stroke (*p* = 0.009). Both moderate and severe stroke had significantly influenced the direct medical cost of stroke, with each costing MYR 295.77 (*p* = 0.010) and MYR 2397.72 (*p* = 0.011), respectively, which were higher costs than for mild stroke. Meanwhile, patients who were discharged from the hospital against medical advice had incurred costs as much as MYR 8618.85 (*p* < 0.001), a greater cost of stroke care than those patients who had died in the hospital.

### 3.3. Cost-Effectiveness Analysis of DOACs in the Prevention of AF-Related Stroke

#### 3.3.1. Calculation of the Cost Effectiveness Ratio (CER) of DOACs (Versus Warfarin) Based on Stroke Severity According to the mRS Score for AF-Related Ischaemic Stroke Patients

The cost-effectiveness ratio (CER) of DOACs versus warfarin is evaluated based on the calculation method as shown below,

Cost-effectiveness ratio (CER) DOACs or warfarin = cost of DOACs or cost of warfarin (MYR)

percentage (%) of patients with mild stroke (mRS score of 0–2) on DOACs or warfarin

Based on the summary of stroke severity percentage in Table 8, therefore,

1. The CER calculation for DOACs based on the percentage of patients with mild degree of stroke (mRS score of 0–2) is as follows:

$$=\frac{\mathrm{Cost}\;\mathrm{of}\;\mathrm{stroke}\;\mathrm{treatment}\;\mathrm{with}\;\mathrm{DOACs}\;\mathrm{per}\;\mathrm{patient}\;\left(\mathrm{MYR}\right)}{\mathrm{Percentage}\;\mathrm{of}\;\mathrm{patients}\;\mathrm{with}\;\mathrm{DOACs}\;\mathrm{with}\;\mathrm{mild}\;\mathrm{stroke},\;\mathrm{mRS}\;0-2\;\left(\%\right)}\;=\frac{\mathrm{MYR}\;2839.73}{64.8}\;\;=\mathrm{MYR}\;43.82$$

2. The CER calculation for warfarin based on the percentage of patients with mild degree of stroke (mRS score of 0–2) is as follows:

$$=\frac{\mathrm{Cost}\;\mathrm{of}\;\mathrm{stroke}\;\mathrm{treatment}\;\mathrm{with}\;\mathrm{warfarin}\;\mathrm{per}\;\mathrm{patient}\;\left(\mathrm{MYR}\right)}{\mathrm{Percentage}\;\mathrm{of}\;\mathrm{patients}\;\mathrm{with}\;\mathrm{warfarin}\;\mathrm{with}\;\mathrm{mild}\;\mathrm{stroke},\;\mathrm{mRS}\;0-2\;\left(\%\right)}\;=\frac{\mathrm{MYR}\;2269.79}{35.2}\;\;=\mathrm{MYR}\;64.48$$

Table 9 shows a comparison of CER and ICER values for both interventions (DOACs vs. warfarin) for AF-related stroke treatment in the study population. Based on the calculation, the CER of MYR 43.82 obtained for DOACs was much lower than the CER for warfarin (MYR 64.48), indicates that NOACs are more cost effective in the treatment of AF related stroke patients in UKMMC.

#### 3.3.2. Calculation of the Incremental Cost-Effectiveness Ratio (ICER) of DOACs (versus Warfarin) Based on the Severity of the Stroke by Using the mRS Score for AF-Related Stroke Patients

Based on the ICER calculation formula:

(1) $$\frac{\mathrm{difference}\;\mathrm{in}\;\mathrm{cost}\;\mathrm{of}\;\mathrm{stroke}\;\mathrm{treatment}\;\mathrm{with}\;\mathrm{DOACs}\;\mathrm{and}\;\mathrm{warfarin}\;\left(\mathrm{MYR}\right)}{\mathrm{difference}\;\mathrm{in}\;\mathrm{percentage}\;\mathrm{of}\;\mathrm{patients}\;\mathrm{with}\;\mathrm{mild}\;\mathrm{stroke}\;\mathrm{on}\;\mathrm{DOACs}\;\mathrm{and}\;\mathrm{warfarin}\;}\;=\frac{\mathrm{MYR}\;2839.73-\mathrm{MYR}\;2269.79}{64.8-35.2}\;=\mathrm{MYR}\;19.25\;(\mathrm{additional}\;\mathrm{cost}\;\mathrm{per}\;\mathrm{additional}\;\mathrm{patient}\;\mathrm{with}\;\mathrm{mild}\;\mathrm{stroke}\;(\mathrm{mRS}\;\mathrm{score}\;\mathrm{of}\;0=2$$

= MYR 19.25 (additional cost per additional patient with mild stroke (mRS score of 0–2)

Based on the ICER value obtained, DOACs were found to be more cost effective than warfarin in stroke treatment among AF patients in UKMMC, with a positive ICER value of MYR 19.25.

## 4. Discussion

In line with previous studies, our study revealed that the overall direct medical cost of stroke was substantial, and that AF-related strokes led to a longer length of stay (LOS), incurred a higher median cost per patient and most AF-stroke patients also experienced moderate to severe stroke episodes. Based on the results of this study, the overall direct medical cost of stroke from 2011 to 2018 was MYR 11,669,414.83, and AF-stroke cost accounted for ~10% of the total direct medical cost for stroke care in those 7 years. In terms of LOS, the median LOS was 5 days for the overall cohort of stroke subjects. These findings are in line with other previous stroke studies, for example, Göz et al. (2017) [33] reported an average LOS of 7 days (IQR: 5–13 days) among stroke patients. An earlier study conducted in similar facility (UKMMC) also reported similar findings with an average LOS in hospital of 6.4 days [16]. Another study, which was also conducted in UKMMC and which investigated the LOS for stroke patients in the same facility over the duration of 2 years, has reported that the median LOS for their stroke subjects was 4 days [17]. However, a hospital-based study in China reported that their average LOS for stroke subjects were much longer, at 26.7 days [34]. This was attributable to the fact that most of the stroke patients’ stay in hospital included their rehabilitation period as well [34]. Because most of the stroke subjects in this study had mild (45.9%) to moderate (36%) stroke, with only 9.6% of cohort experienced severe stroke, it explains why the median LOS for our study period tend to be shorter.

For our study, the median cost of stroke treatment for overall stroke patients was substantial, at MYR 2269.79 per patient per admission. This accounted for 5% of the country’s per capita GDP (amounting to MYR 46,645.54), as per 2019 estimates by the International Monetary Fund (IMF). However, our direct medical cost for the overall stroke subjects was smaller compared to previous studies. In a Singaporean study, Chow et al. (2010) [35] reported an average cost of SGD 6783.00 among stroke patients at the Singapore General Hospital. The difference was probably due to the additional cost of providing inpatient rehabilitation, which was included in the Singapore study, hence increasing the overall cost of stroke treatment, apart from variations in healthcare financing systems, and differences in clinical practice and research design between these studies. Ng et al. (2015) reported a higher mean direct medical cost of SGD 12,473.70 compared to our study [36]. This could be explained due to the different types of costing methodology applied in both studies. The case mix tariff used in this study was the lumpsum cost based on the top-down costing method as opposed to bottom-up methodology employed in the other study. In top-down costing, results are in average unit costs per patient, based on the allocated cost to the final cost centre, whereas bottom-up micro-costing leads to patient specific unit costs. Although top-down costing gives adequate accuracy, bottom-up micro-costing is more accurate in the hospital setting, since all activities must be identified and resources used associated with it are recorded [37]. When comparing our study to Ng et al. (2015), the costing analysis involved in this study was only based on a single centre, as compared to the latter which enrolled all patients who received care in any of the National Healthcare Group (NHG) institutions (which consists of hospitals, national centre, primary care clinics and specialty institutes).

There was a difference in the length of hospital stay and the direct medical costs involved for the AF-related stroke and non-AF-related stroke subgroups in this study. AF-related stroke patients had longer LOS in the hospital compared to patients without AF (6 ± 4.2 days versus 5 ± 2.9 days, respectively). A higher median cost per patient per admission compared to non-AF related stroke patients was also observed (MYR 2839.73 versus MYR 2338.40) which is incongruent with the findings of other previous studies [12,13,14]. However, the total direct medical cost incurred by the AF stroke cases in our study was much lower (MYR 1,219,181.36) compared to the non-AF stroke cases (MYR 10,450,233.47). In contrast, Ali et al. (2015) reported a much higher total direct cost of EUR 11,799, which can be explained by the fact that the AF stroke cases in their study was approximately 60% of the total participants recruited and also by the bottom-up costing approach employed, which was more accurate as it includes all aspects of patient’s activity during hospitalisation [14]. The proportion of AF stroke patients in our study was much lower, at 9.3% from our total participant cohort.

This study found important determinants for the total direct cost of stroke in UKMMC. The types of strokes (AF-related stroke and non-AF related stroke), and the patients’ LOS in the hospital were two major cost drivers. The LOS of stroke patients in the hospital has become a significant cost driver (*p* < 0.001) in this study, in which the longer the patients’ LOS, the higher the cost of treatment the patient will have to bear. The median direct medical cost of stroke for patients who stayed more than 7 days in the hospital was higher, amounting to MYR 2839.73, compared to other groups of patients with LOS of ≤3 days and between 4–7 days, with MYR 2269.79, respectively. This is consistent with the findings of other studies, which also reported that the cost of stroke care is strongly correlated with the length of hospital stay [14,38]. When making a comparison in terms of the cost of care between AF related stroke and non-AF stroke, the median direct medical cost incurred for AF related stroke patients was MYR 2839.73, as compared to MYR 2269.79 for the non-AF related stroke patient. It indicates that the presence of AF will give rise to a higher cost of care among stroke patients. Significantly, AF-related stroke consumed about MYR 274.57, which represents higher resources per stroke patient per admission than the non-AF related stroke (*p* = 0.013). This agrees with previous studies which reported that, due to severe functional deficits and an increase in the rate of complications that are often associated with AF related stroke patients, it will not only lead to higher mortality rates but also increase the complexity of treatment and, extensive use of medical resources, and thus will further prolong the patients’ LOS in the hospital [10,13,14]. The median LOS of AF-related stroke patients in the hospital found in this study was 6 days, slightly longer compared to non-AF-related stroke (5 days). Regression analysis also found that the LOS for AF-related stroke patients in the hospital was 1.3 days, longer than non-AF stroke patients (*p* = 0.019), which were in congruent with earlier studies [14,39]. 

For the sub-group of AF-related stroke patients, variables such as the severity of stroke (using mRS score), sub-types of strokes (ischemia, haemorrhagic, and unspecified), and patients’ discharge disposition status were the factors that significantly affect the cost of stroke treatment. The median cost of treatment for AF related stroke patients was the highest for severe stroke (mRS score of 5) with MYR 3101.52, followed by moderate stroke (MYR 2839.73), and mild stroke (MYR 2269.79). Based on the results of regression analysis, AF-related stroke patients with severe stroke (mRS score of 5) had a treatment cost of MYR 2397.72, higher than those with mild stroke (mRS score of 0–2) (*p* = 0.011). Previous studies have stated that the direct medical cost of stroke was strongly correlated with stroke severity [16,40,41]. With the increasing severity of a stroke, the LOS of stroke patients will be prolonged in the ward. The treatment involved will also be more difficult and complicated, and longer monitoring was needed to ensure the stability of the patient’s condition before they were allowed for discharge from the hospital [16]. 

The discharge status of the AF-stroke patients also had influence on the direct medical cost. The group of AF related stroke patients who were discharged from the hospital against medical advice had a higher cost of care at MYR 8618.853 (*p* < 0.001) than those who had died. A possible explanation for this is because these patients are often associated with various comorbidities and worse health outcomes, and likely result in the incremental cost for treatment as it involves the use of more medical resources and a longer period of hospital stay [42]. They also tend to have psychosocial issues and poorer social support. As a result, they will probably opt for early discharge to reduce the cost burden of stroke they have to bear. 

The cost of care among AF related stroke patients was also compared between the different types of the oral anticoagulant (DOACs vs. warfarin) that have been prescribed to during their stroke treatment. Although the median direct medical costs of patients in UKMMC with the use of DOACs were higher compared to warfarin (MYR 2839.73 versus MYR 2269.79), patients using DOACs had shorter LOS in the hospital, which was 5 days compared to 7 days for patients who were on warfarin. There were previous studies which have reported that the average LOS for AF patients in the hospital with the use of DOACs is shorter (4.5 days) compared to 5.3 days for the group of patients receiving warfarin [43]. These findings were supported by similar studies which have also reported that the direct medical costs of stroke were reduced with the use of DOACs instead of warfarin for the treatment of AF-related stroke patients. Given the potential cost savings that DOACs could offer, since it may reduce the risk of bleeding and prevent strokes, these agents would be a promising alternative for stroke prevention treatment.

Over half of the patients in the AF group had a high risk of stroke with a CHADS2 score of 2 or greater. Such a score indicated that oral anticoagulant therapy for stroke prevention in these patients should be considered, unless contraindicated [44]. In this study, most patients who were treated with DOACs post-stroke event during hospitalisation received dabigatran 150 mg (25%), followed by rivaroxaban 20 mg (17%) and apixaban 5 mg (12.5%). It is also noteworthy to highlight that despite the benefits of oral anticoagulation in the AF group, prior to index stroke, most AF patients (80.2%) in our study were not anticoagulated. Only approximately 20% were baseline oral anticoagulants (DOACS 7.3%, warfarin 12.2%). Although there was a rise in the rate of oral anticoagulant prescriptions after the stroke event, it is still relatively low. Such a pattern could possibly be explained by a number of factors, mainly due to the presence of contraindications to anticoagulant drugs in these patients, in view of their high risk of bleeding, poor comorbid conditions, poor compliance with the medication, poor social support, and advanced age. Many studies have addressed and validated these contributing factors that lead to under-prescription or discontinuation of oral anticoagulants [45,46,47,48,49,50]. In addition to that, a number of patients simply were not keen on being prescribed warfarin for logistical reasons because of the requirement for regular monitoring of the drug, the inconvenience of dose adjustments, and fear of complications [51]. The higher price tag of DOACs could also be one of the deciding factors in the choice of treatment for stroke prevention among patients. Overall, 72.9% of the 181 patients who were not anticoagulated during their hospitalisation received antiplatelet therapy, while the remaining 27.1% did not. The majority of those who did not have any contraindications but were not anticoagulated were either prescribed with antiplatelets, such as aspirin (84.1%), or clopidogrel (15.9%). They were also on medications such as statins, angiotensin converting enzyme inhibitors (ACEI), and calcium channel blockers for the management of their various underlying comorbidities. Another Malaysian stroke study also highlighted that less than half of their AF stroke subjects received anticoagulant therapy [52]. Similarly, a hospital-based analysis of AF patients in Kuala Lumpur also revealed that only 16% of AF patients were on warfarin prior to hospital admission [53]. A low level of anticoagulation seen in local studies (including ours) needs to be explored and urgently reflect the need for further improvement in detection and monitoring of AF treatment in the local community. 

To the best of our knowledge, this study was the first to analyse the cost-effectiveness of DOACs in comparison with warfarin in AF-related stroke patients in Malaysia. Any intervention that has been compared with a lower CER value indicates that it is more cost-effective to be adopted as the best treatment to achieve a specific target outcome [30,32,54]. The incremental cost-effectiveness ratio (ICER) is more important than the value of CER in determining the cost-effectiveness of an intervention from the economic point of view [30,54]. This is because ICER provides more information to how much patients are paying for each extra unit of effectiveness achieved by undertaking the new intervention. This study demonstrated that DOACs are more cost-effective than warfarin for the treatment of AF-related ischaemic stroke patients, which is consistent with other cost-effectiveness studies of DOACs against warfarin [55,56,57]. The ICER value for this study is MYR 19.25, which shows that DOACs are more cost effective than warfarin. In contrast, a study by Dilokthornsakul et al. (2018) [58] reported a contrary finding that all DOACs are not cost-effective compared to warfarin among AF patient populations in Thailand. Nevertheless, they suggested that of all the DOACs, apixaban may potentially be a cost-effective option, if the drug acquisition cost of the drug is lower than the current market price. Considerations should also be given to the quality and cost incurred for the establishment and maintenance of anticoagulation clinics for stroke patients monitoring who have been using warfarin. With the use of DOACs, the need for anticoagulation clinics may be reduced, and this will generally save more costs on the healthcare system. The findings on the cost-effectiveness analysis of DOACs found in this study was probably influenced by the method used for the calculation of ICER. Many of previous cost-effectiveness studies on DOACs were conducted using a detailed economic analysis based on Markov modelling that is constructed to project the lifetime costs and quality-adjusted life years (QALYs) obtained with the use of DOACs compared with warfarin [55,56,59]. Meanwhile, the method used for the ICER calculation in this study was not based on the Markov economic model. Instead, we calculated the ICER using the ratio of the difference between the costs incurred for DOACs and warfarin, divided by the difference in the severity outcome of patients (in terms of percentage) on DOACs versus warfarin. Since only 45% of AF patients were anticoagulated (either DOACs or warfarin), this explains why our ICER value was smaller compared to other studies.

To conclude on the cost-effectiveness of any health care interventions, the ICER value is generally compared with a reference value, which is the ICER or cost effectiveness (CE) threshold. The World Health Organization (WHO) has made a generic recommendation for CE threshold in developing countries to take the value of 1 to 3 times the gross domestic product (GDP) per capita of the country [60]. However, in Malaysia, there is no such definite threshold value [31]. The CE threshold signifies the willingness to pay (WTP) per quality-adjusted life year (WTP/QALY) gained, and is a crucial part of decision making involving economic evaluation [61]. As stated by Lim et al. (2017) [31], the estimated CE threshold value that ranged from MYR 12,810 to MYR 22,840 (~USD 4000–USD 7000) could serve as a universal threshold for Malaysia. In the context of this study, the ICER value of MYR 19.25 obtained for DOACs is lower than the gross domestic product (GDP) per capita of the country (according to 2019 estimates) and the CE threshold value proposed by Lim et al. (2017) [31], proving that DOACs are very cost-effective in AF related stroke prevention treatment in Malaysia.

However, this study is not without limitations. First, the estimation of direct medical cost of stroke was based on the case mix tariff which represents the lumpsum cost of all components of the cost centres in UKMMC, using a top-down costing approach. Although the top-down costing method provides reasonable accuracy and has been widely used for hospital services in developing countries, it does not identify all costs directly associated with a particular activity, especially at the patient level. The best way to estimate patient’s direct medical cost is to use the top-down costing and combine it with other methods (such as bottom-up approach) if necessary, to produce a more comprehensive data set. Second, the calculation of CER and ICER of DOACs were based on patients’ baseline mRS score, during their ward admission. A comparison of a 3-month stroke disability, using the same mRS score, should be made for these patients following their hospital discharge, to increase the accuracy of the study findings. Another limitation of the study is the nature of the study itself. As this was a retrospective study, the information that was based on whatever available data from the patients’ database (from either patient case notes or hospital records) were reliable, but we cannot deny the possibility of recall bias (such as dietary history or specific drug prescriptions) from the patient’s side, or the fact that some important information was simply not available or recorded. The fact that we must rely on others for accurate record keeping may introduce some biases and this may affect the quality of the analyses, and the study findings. Since the secondary data used in this study was obtained only from a single centre, it may not be representative of all Malaysian hospitals. Lack of prior research study on the cost effectiveness of DOACs in the prevention of AF-related stroke is also another limitation, as it may not allow us to generalise our findings towards the Malaysian population as a whole. 

## 5. Conclusions

This study provides information on the direct medical cost of stroke, with a specific emphasis on the costs incurred by AF-related stroke patients. Despite the limitations, our study also provides a glimpse into the cost-effectiveness profile of DOACs as a treatment alternative among AF-related stroke patients in Malaysia. Although the length of stay for stroke patients in this study was shorter compared to the other studies, the cost of care for these patients was significant. The direct medical cost of stroke in this study was found to be substantial, which contributed to 5% of the per capita gross domestic product (GDP) of the country, as per 2019 estimates by International Monetary Fund. Factors affecting the direct medical cost of stroke include the types of strokes (AF versus non-AF), stroke subtypes, stroke severity level, and patients’ discharge disposition status. Based on patients’ stroke severity (using the mRS score), DOACs were found to be cost effective compared to warfarin in the treatment of AF-related stroke in UKMMC, although this finding may not be generalisable to the whole stroke patient population in Malaysia. Comprehensive studies that evaluate more components of cost, using both health care providers’ and patients’ perspective and combining both top-down and bottom-up costing method, are required to estimate the economic burden of stroke in this country. A bigger sample drawn from more diverse health institutions would also increase the representativeness of the study results. More comprehensive studies on the cost-effectiveness of DOACs for stroke treatment among the Malaysian population should be emphasised for the benefits of a results comparison and in view of the potential cost savings that DOACs could produce.

## Figures and Tables

**Table 1:** Characteristics of all study participants (n = 3689).

Characteristics	Subgroups	n (%)
Stroke types	AF-related stroke	344 (9.3)
	Non-AF-related stroke	3345 (90.7)
Mean (SD) length of stay, LOS (days)	5 ± 3.4	-
LOS (grouping)	≤3 days	1156 (31.3)
	4–7 days	1556 (42.2)
	>7 days	977 (26.5)
Mean age (SD)	64 ± 14.6	-
Age group (years)	<50	629 (17.1)
	50–59	751 (20.4)
	60–74	1509 (40.9)
	75 and above	800 (21.7)
Gender	Male	2223 (60.3)
	Female	1466 (39.7)
Stroke subtype	Ischemic stroke	2305 (62.5)
	Haemorrhagic stroke	613 (16.6)
	Unspecified stroke	771 (20.9)
Level of stroke severity (mRS score)	Mild (score of 0–2)	1695 (45.9)
	Moderate (score of 3–4)	1329 (36.0)
	Severe (score of 5)	354 (9.6)
	Died (score of 6)	221 (6.0)
	Unable to determine	90 (2.4)
Main comorbidities	Diseases of circulatory system	1911 (51.8)
	Diabetes	1029 (27.9)
	Diseases of respiratory system	409 (11.1)
	Renal diseases	159 (4.3)
	Others	181 (4.9)
Causes of mortality	Massive infarct	38 (17.2)
	Massive bleed (ICH, etc.)	141 (63.8)
	GIT bleed (UGIB, etc.)	1 (0.5)
	Stroke associated pneumonia	12(5.4)
	Recurrent stroke	12 (5.4)
	Sepsis	2 (0.9)
	Others (Cardiac complications; Metabolic cause, etc.)	15 (6.8)
Discharge disposition status	Home	3422 (92.8)
	Transfer	1 (0)
	Against medical advice	15 (0.4)
	Died	221(6.0)
	Others	30 (0.8)

Abbreviations: ICH (intracranial haemorrhage), GIT (gastrointestinal tract), UGIB (upper gastrointestinal bleed), mRS (modified Rankin score).

**Table 2:** Characteristics of AF-related stroke patients (n = 344).

Variables	Sub-Groups	n (%)
Mean LOS (SD), days	6 ± 4.2 days	-
Length of stay (LOS)	≤3 days	56 (16.3)
	4–7 days	144 (41.9)
	>7 days	144 (41.9)
Mean age (SD)	71 ± 9.8	-
Age group (years)	<50	14 (4.1)
	50–59	38 (11.0)
	60–74	159 (46.2)
	75 and above	133 (38.7)
Gender	Male	176 (51.2)
	Female	168 (48.8)
Stroke subtype	Ischemic stroke	252 (73.3)
	Haemorrhagic stroke	20 (5.8)
	Unspecified stroke	72 (20.9)
Stroke severity (mRS score)	Mild (score of 0–2)	80 (23.3)
	Moderate (score of 3–4)	173 (50.3)
	Severe (score of 5)	49 (14.2)
	Died (score of 6)	30 (8.7)
	Unable to determine	12 (3.5)
Main comorbidities	Diseases of circulatory system	179 (52.0)
	Diabetes	88 (25.6)
	Diseases of respiratory system	12 (3.4)
	Renal diseases	25 (7.2)
	Others	40 (11.7)
Ward	Medical	246 (71.5)
	Surgery	27 (7.8)
	General Intensive Care Unit (ICU)	3 (0.9)
	Stroke Care Unit (SCU)	58 (16.9)
	High Dependency Ward (HDW)	1 (0.3)
	Others (Staff ward, Coronary rehabilitation ward)	9 (2.6)
Discharge Disposition Status	Home	305 (88.7)
	Against medical advice	5 (1.5)
	Died	30 (8.7)
	Others	4 (1.2)
CHADS2 Score	Score of 0	32 (9.3)
	Score of 1	124 (36.1)
	Score of 2 or more	188 (54.7)

**Table 3:** Oral anticoagulant treatment profile for AF-related stroke patients (n = 344).

Variables	Sub-Groups	n (%)
Baseline anticoagulant therapy (before index stroke event)	None	276 (80.2)
	DOACs	25 (7.3)
	Warfarin	42 (12.2)
	Both DOACs and warfarin	1 (0.3)
Baseline DOACs (before index stroke event) (n = 25)	Dabigatran 110 mg	8 (32.0)
	Dabigatran 150 mg	4 (16.0)
	Rivaroxaban 15 mg	5 (20.0)
	Rivaroxaban 20 mg	3 (12.0)
	Apixaban 2.5 mg	2 (8.0)
	Apixaban 5.0 mg	3 (12.0)
Anticoagulant therapy after the stroke event (in the ward)	None	181(52.6)
	DOACs	88 (25.6)
	Warfarin	67 (19.5)
	Both DOACs and warfarin	8 (2.3)
DOACs use after the stroke event (in the ward) (n = 88)	Dabigatran 110 mg	19 (21.6)
	Dabigatran 150 mg	22 (25.0)
	Rivaroxaban 15 mg	7 (8.0)
	Rivaroxaban 20 mg	15 (17.0)
	Apixaban 2.5 mg	7 (8.0)
	Apixaban 5.0 mg	11 (12.5)
	Dabigatran + Rivaroxaban	3 (3.4)
	Dabigatran + Apixaban	4 (4.5)

**Table 4:** Length of stay and direct medical cost of stroke for total stroke cases, sub-group of AF-related stroke cases, and non-AF related stroke cases.

Variables	Total Stroke Cases (n = 3689)	AF-Related Stroke Cases (n = 344)	Non-AF Related Stroke Cases (n = 3345)	*p* Value *
Mean LOS, days (SD)	5 ± 3.4	6 ± 4.2 days	5 ± 2.9	0.002
Total direct medical cost of stroke (MYR)	11,669,414.83	1,219,181.36	10,450,233.47	0.001
Median cost per patient per admission MYR (IQR)	2269.79 (2269.79–2924.96)	2839.73 (2269.79–3101.52)	2338.40 (2110.82–2876.16)	<0.001
Median cost per inpatient day, MYR (IQR)	453.96 (321.20–362.45)	473.29 (289.11–412.43)	467.68 (365.23–422.98)	<0.001

Abbreviations: MYR (Malaysian Ringgit), IQR (interquartile range); * Kruskal–Wallis test

**Table 5:** Length of stay, median cost and median mRS score for AF ischaemic stroke patients who were on oral anticoagulants during hospitalisation.

	DOACs (n = 88)	Warfarin (n = 67)	*p* Value *
Median cost, MYR (IQR)	2839.73 (2269.7–3101.52)	2269.76 (2109.72–3607.56)	0.069
Median LOS, days (IQR)	5.00 (4.00–8.25)	7.00 (5.00–11.00)	0.121
Median mRS score (IQR)	3.00 (2.00–4.00)	4.00 (2.00–4.00)	0.538
Total direct medical cost (MYR)	271,504.41	188,068.14	0.087
Percentage of patients with mild degree of stroke (mRS score of 0–2) during hospitalisation (%)	64.8	35.2	-

* Mann-Whitney U test.

**Table 6:** Standard multiple linear regression analysis for cost of stroke care (MYR) for total stroke patients (n = 3689).

Independent Variables		Coefficient (SE)	t Value	*p* Value
Constant		2453.256 (774.392)		
Stroke types	AF-related stroke (vs. non-AF related stroke)	274.574 (210.965)	1.302	0.013 *
Length of hospital stay (LOS)	4–7 days (vs. ≤3 days)	187.17 (348.04)	0.538	0.197
	>7 days (vs. ≤3 days)	2158.01 (2679.73)	0.801	0.001 *

* *p* value significant at 0.05.

**Table 7:** Standard multiple linear regression analysis for the cost of stroke care (MYR) for AF-related stroke patients.

Independent Variables		Coefficient (SE)	t Value	*p* Value
Constant		3224.149 (2248.759)		
Stroke sub-types	Unspecified stroke (vs. ischemic stroke)	−351.790 (557.398)	−0.631	0.689
	Haemorrhagic stroke (vs. ischemic stroke)	1514.265 (638.068)	2.373	0.009 *
Stroke severity level (mRS score)	Moderate (score of 3–4) (vs. mild (score of 0–2))	295.774 (825.464)	0.358	0.010 *
	Severe (score of 5) (vs. mild (score of 0–2))	2397.724 (921.715)	2.601	0.011 *
Patients’ discharge disposition status	Home (vs. “died”)	104.484 (1204.216)	0.087	0.931
	Against medical advice (vs. “died”)	8618.853 (2370.864)	3.635	<0.001 *

* *p* value significant at 0.05.

**Table 8:** Percentage of AF related stroke patients with different stroke severity level (mRS score) based on the types of oral anticoagulant therapy received in the ward (DOACs vs. warfarin).

	AF Related Stroke Severity Level (mRS score)					Total, n (%)
Types of Oral Anticoagulant	Mild (Score 0–2)	Moderate (Score 3–4)	Severe (Score 5)	Died (Score 6)	Unable to Determine	
DOACS, n (%)	35 (64.8)	42 (52.5)	6 (54.5)	0	5 (71.4)	88 (56.8)
Warfarin, n (%)	19 (35.2)	38 (47.5)	5 (45.5)	3 (100)	2 (28.6)	67 (43.2)
Total, n (%)	54 (100)	80 (100)	11 (100)	3 (100)	7 (100)	155(100)

**Table 9:** Summary of CER for DOACs (vs. warfarin) based on the percentage of patients. with mild degree of stroke (mRS score of 0–2).

Intervention (Oral Anticoagulant Therapy)	Median Direct Medical Cost of AF-Related Stroke (*“per patient”*) (MYR)	Outcome (Percentage of Patients with Mild Degree of Stroke (mRS Score of 0–2) (%)	Median Incremental Cost per Patient (MYR)	CER (MYR)	ICER
DOACs	2839.73	64.8	569.94	43.82	19.25
Warfarin	2269.79	35.2	-	64.48	-

## Data Availability

Not applicable.
